# A Comparison Study on the Characteristics of Nanofibrils Isolated from Fibers and Parenchyma Cells in Bamboo

**DOI:** 10.3390/ma13010237

**Published:** 2020-01-06

**Authors:** Xiaofeng Zhang, Hanxiao Huang, Yan Qing, Hankun Wang, Xingong Li

**Affiliations:** 1 College of Materials Science and Engineering, Central South University of Forestry and Technology, Changsha 410004, China; zhangxf@icbr.ac.cn (X.Z.); huanghx@icbr.ac.cn (H.H.); qingyan0429@163.com (Y.Q.); 2 Institute of New Bamboo and Rattan Based Biomaterials, International Center for Bamboo and Rattan, Beijing 100102, China; 3 SFA and Beijing Co-built Key Lab for Bamboo and Rattan Science and Technology, Beijing 100102, China

**Keywords:** bamboo, parenchyma cells, lignocellulose nanofibrils (LCNFs), cellulose nanofibrils (CNFs)

## Abstract

In this study, bamboo fibers and parenchyma cells were separated by a physical water-medium method. To compare the characteristics of nanofibrils from these two types of cells, lignocellulose nanofibrils (LCNFs) and cellulose nanofibrils (CNFs) were prepared by different processes. Atomic force microscopy analysis revealed that both fibers and parenchyma cells can be separated into individual fibrils after grinding three times. However, LCNFs had a diameter of 20–40 nm, which was larger than that of CNFs (10–20 nm). Additionally, the films prepared from LCNFs had lower tensile strength, but higher hydrophobicity compared with those from CNFs. X-ray diffraction analysis and tensile test of the films showed that the nanofibrils isolated from fibers and parenchyma cells had similar crystallinity and mechanical properties. This study shows a promising application of bamboo parenchyma cells, which are usually discarded as waste in the processing of bamboo products, in the preparation of nanofibers.

## 1. Introduction

Cellulose is synthesized by a wide range of organisms, including all green plants, certain bacteria, fungi, and even some animals, and thus is one of the most abundant renewable resources on earth. Cellulose nanofibrils (CNFs), which have a diameter of 10–50 nm and a length of several micrometers [1], have been prepared from lignocellulose biomass. Due to their exceptional mechanical properties and high aspect ratio, they have attracted much attention for applications in films [2], composite materials [3] and electronics [4].

Currently, CNFs are generally prepared by a process involving chemical pretreatment and mechanical fibrillation. Different pretreatments can be used before the mechanical fibrillation process to reduce energy consumption and endow the CNFs with new properties [5]. Cellulose is found in the plant cell wall as nanofibrils assembled into microfibril bundles chemically bound to hemicellulose and lignin [6]. Lignin is usually removed by chemical pretreatment in most methods used in research on CNF production. However, some studies have reported that fibrillated material with residual lignin could be a good alternative to bleached fibers in the production of lignocellulose nanofibrils (LCNFs), particularly considering with advantages of higher hydrophobicity, good thermal properties, high yields and more environmental friendliness [7,8]. CNFs are widely popular, but the high content of free hydroxyl groups makes them highly hydrophilic, which results in a high absorption of moisture and incompatibility with many hydrophobic thermoplastics [9]. On the other hand, lignin can prevent the free hydroxyl groups on CNFs from forming hydrogen bonds with water molecules, and thereby enhance the water barrier properties [10]. Recent studies have demonstrated that composites reinforced with LCNFs have much better mechanical properties than those reinforced with a similar amount of CNFs [11,12]. This was attributed to the residual lignin in LCNFs which could act as a compatibilizer to hydrophobic polymers and potentially improve their dispersion in the composite system. Furthermore, the production of LCNFs only requires a simplified preprocessing step to improve the yield and reduce the cost and environmental impact [13].

As an abundant lignocellulosic material with high cellulose content, bamboo is an important raw material for the production of nanocellulose. Recently, Lu reported a novel method to prepare LCNFs directly from bamboo chips [14]. Junior compared the characteristics of TEMPO-oxidized cellulose nanofibers and the nanopapers prepared from bamboo, softwood and hardwood pulps [15]. At the cellular level, bamboo culm wall consists of fibers in vascular bundles and many parenchyma cells in the matrix, which act as a transport system and food storage organs, respectively. Additionally, there are great differences in morphology, chemical composition and microfibril orientation between fibers and parenchyma cells. However, currently, in the processing of bamboo products, parenchyma cells are not considered to be conducive to good quality and are usually discarded as waste. Thus, finding useful applications for parenchyma cells will not only be beneficial for improving the comprehensive utilization rate of bamboo, but also for alleviating the environmental pollution associated with the disposal of bamboo wastes. Besides their various functions in plants, parenchyma cells also have great potential for application in the production of nanocellulose-like fibers. The successful production of cellulose nanocrystals from both parenchyma and the vascular bundle of oil palm trunks was previously demonstrated by Lamaming [16]. Some other studies also reported that CNF films prepared from bamboo fibers and parenchyma cells had similar mechanical properties [17,18]. However, in the above-mentioned studies, the bamboo tissue samples were homogenized into single cells by the removal of lignin, and then separated into fibers and parenchyma cells. To the best of our knowledge, the preparation of LCNFs from fibers and parenchyma cells of bamboo has not been reported to date.

Due to the low utilization of bamboo parenchyma cells in production, the main objective of this study is to explore the potential of parenchyma cells for preparing nanocellulose. In order to compare the LCNFs and CNFs isolated from bamboo fibers and parenchyma cells, we have chosen mature Moso bamboo (*Phyllostachys edulis*) as raw material. Bamboo culms were crushed into powder and then homogenized in deionized water to obtain fibers and parenchyma cells. LCNFs and CNFs were prepared after different chemical pretreatment followed by the same mechanical grinding procedure. The prepared corresponding films were analyzed to evaluate their mechanical properties.

## 2. Materials and Methods 

### 2.1. Materials

**Figure 1:**
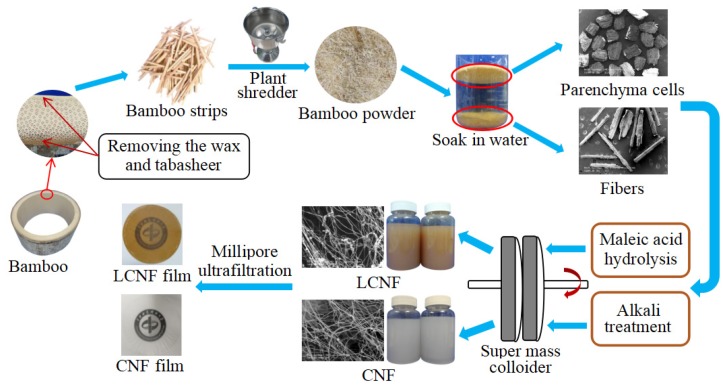
A schematic flow diagram of bamboo fibers and parenchyma cells for producing CNF (cellulose nanofibril) and LCNF (lignocellulose nanofibril) film.

Mature 4-year old culms of Moso bamboo (Phyllostachys edulis) were collected from Hangzhou Zhejiang Province, China. After removing the bamboo wax and tabasheer, the culms were cut into smaller chips of size 4–5 cm and then ground into powder before sieving through screens of 30 and 60-mesh size (Figure 1).

### 2.2. Separation of Fibers and Parenchyma Cells

Fibers and parenchyma cells were separated by a physical water-medium method. Bamboo powder samples obtained with 30–60 mesh sieves were immersed in deionized water for 2 min. Parenchyma cells will float on the surface, while fibers immediately sink to the bottom due to their difference in density. Thus, we were able to quickly retrieve the parenchyma cells, and the separation was achieved simply and efficiently. The separated fibrous fibers and rectangular parenchyma cells are shown in Figure 1. These images confirmed that the fibers and parenchyma cells had been successfully separated from the bamboo culms.

### 2.3. Isolation of Cellulose Nanofibrils and Lignocellulose Nanofibrils

Air-dried fibers and parenchyma cells were acid hydrolyzed with a 60 wt% maleic acid solution at 120 °C for 180 min, using a liquor to solid ratio of 15:1 (L/kg) [19]. The hydrolysate residues were extensively washed with hot water and the washed residues, which were stored in a cool place, were termed as lignin cellulosic solid residues (LCSRs).

Additionally, cellulosic solid residues (CSRs) were prepared as follows. Air-dried fibers and parenchyma cells were bleached with an acidified sodium chlorite solution at 75 °C for 1 h, and then this process was repeated five times. Subsequently, the samples were treated with 2 wt% KOH at 90 °C for 2 h, followed by treatment with the acidified sodium chlorite solution for 1 h. Afterwards, the samples were treated with 5 wt% KOH at 90 °C for 2 h, followed by treatment with 1 wt% HCl solution at 80 °C for 2 h [20]. Eventually, after thoroughly washing the residues with deionized water, the CSRs were obtained.

The LCSR and CSR samples at 1 wt% were separated into individual fibrils using a MKCA6-5J, Supermass Colloider (Masuko Sangyo Co., Ltd., Kawaguchi, Japan) at 1500 rpm/min. The samples were ground once with a grindstone at the zero position and then 3 times with a clearance gauge of −100 µm. The ground LCSR and CSR samples were named lignocellulose nanofibrils (LCNFs) and cellulose nanofibrils (CNFs), respectively. The abbreviations of the products obtained at each stage of the sample preparation are listed in Table 1.

### 2.4. Preparation of LCNF and CNF Films

Films were prepared with 250 mL of a diluted (0.1 wt%) LCNF/CNF suspension that had been dispersed with a magnetic stirrer for 24 h to remove bubbles [21]. The suspension was then filtered using a Buchner funnel with polyvinylidene difluoride (PVDF) 0.22-μm pore size membranes. The suspension was dehydrated on individual films and placed between two PVDF membranes and blotting papers. Then, the films were pressed at 345 kPa for 10 min and ultimately placed under a weight of 20 kg to dry at room temperature for 5 days.

### 2.5. Characterization

The chemical composition of the raw materials and treated samples was determined. Klason lignin and α-cellulose were analyzed by NaOH and H_2_SO_4_ hydrolysis, respectively, and the hemicellulose was determined by subtracting the α-cellulose content from the holocellulose content.

The chemical functional groups of the raw materials and treated solid residues were analyzed by Fourier-transform infrared (FTIR) spectroscopy using a Nicolet Nexus 670 FTIR spectrometer (Thermo Fisher Scientific, Waltham, MA, USA). All the samples were freeze-dried and ground into powder.

The morphology of the LCSR and CSR samples was examined by scanning electron microscopy (SEM) using a Philips XL30 ESEM-FEG environmental scanning electron microscope system (Philips B.V., Eindhoven, The Netherlands) at 7 kV. All the samples, at a concentration of 0.01 wt%, were freeze-dried and then sputter-coated with a thin gold layer to provide enough conductivity.

The morphology of the LCNF and CNF samples was also studied by atomic force microscopy (AFM) performed on an ICON atomic force microscope (Bruker AFM, Santa Barbara, CA, USA) in advanced tapping mode. The samples were diluted to a solid concentration of 0.01 wt% and then deposited on a clean mica substrate to air dry at room temperature.

The crystallinity (CrI) of the LCSR/CSR samples and LCNF/CNF samples was determined by wide-angle X-ray diffraction on a PANalytical X’Pert PRO X-ray diffractometer (PANalytical B.V., Almelo, The Netherlands) in the range of 5–40°. All the samples, at a solid content of 0.2 wt%, were freeze-dried and then pressed into pellets at 180 MPa. The CrI was calculated according to the Segal method. The average values of the CrI were calculated from five samples.

The thermogravimetric analysis (TGA) of the samples obtained at different stages was performed on a Q500 thermogravimetric analyzer (Q500, TA Instruments, New Castle, DE, USA). The dried samples (8–10 mg) were heated in platinum crucibles from ambient temperature to 600 °C at a rate of 10 °C /min under a 20 mL/min high purity nitrogen stream.

The surface wettability of the LCNF and CNF films was measured by static contact angle analysis using an OCA20 contact angle goniometer (Dataphysics Instruments, GmbH, Filderstadt, Germany). The volume of the water droplet, approximately 3 µL, was accurately controlled by a microsyringe. The water contact angle (WCA) was recorded by placing the droplet on the film for different times at five different positions.

The mechanical properties of the films were measured according to the ASTM standard method. The tensile tester was equipped with a 50-N load cell at a crosshead speed of 1 mm/min. The specimens were dumbbell-shaped and were placed in the room for 48 h before testing. The tensile strength and elastic modulus were obtained from the stress–strain curves. Three films were prepared for each sample and five tensile specimens were obtained from each film and used for measurements. The data reported in this study are the average values of these specimens.

## 3. Results

### 3.1. Yields and Chemical Composition

**Figure 2:**
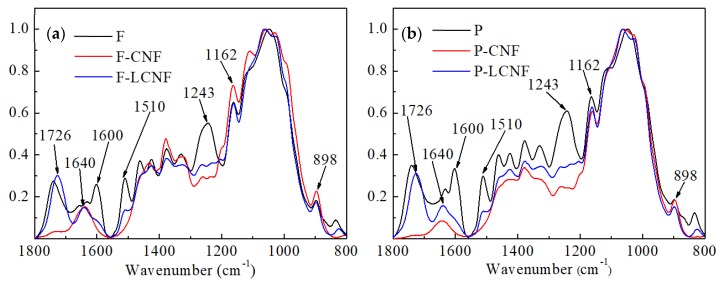
FTIR spectra of samples at different stages from fibers (**a**) and parenchyma cells (**b**).

FTIR spectra were obtained to characterize the changes in chemical structure of the analyzed samples after various treatment. The spectra of the LCSR and CSR samples displayed in Figure 2 clearly show the adsorption peak associated with the vibration of water molecules at around 1640 cm^−1^, which was difficult to completely remove due to the cellulose–water interaction [22]. The absorption bands observed at 1162 and 898 cm^−1^ were attributed to typical cellulose structures and appeared in all the samples [23,24], indicating that the chemical pretreatment did not destroy the cellulose present in the raw materials. The bands at 1510 and 1461 cm^−1^ were ascribed to the aromatic rings in lignin [25,26] and were detected only in the raw materials and LCSR samples, demonstrating that the CSR samples were lignin-free. The peak at 1242 cm^−1^ was assigned to the CO of the glucuronic acid unit in hemicellulose [27]. The weak peak present in the LCSR samples, but almost absent in the CSR samples, may be due to the low ionization degree of maleic acid that weakened its hydrolysis capacity, and thus could only partially hydrolyze the hemicellulose. An additional peak is present at 1726 cm^−1^ in the spectrum of the LCSR samples but not in those of the feed fibers and CSR samples. Such a peak corresponds to the C=O stretching vibrations of the carbonyl group from cellulose esterification by maleic acid [19], and one of the carboxyl groups in the maleic acid reacted with cellulose to form ester groups on the LCNFs [28,29].

The chemical composition and yield from different materials and chemical treatment are described in Table 2. These results reveal that fibers and parenchyma cells obtained by the same chemical pretreatment had a similar chemical composition. The content of α-cellulose in the CNF samples from fibers and parenchyma cells was 84.8 and 84.5%, respectively. In addition, the LCNF samples from these two types of cells also retained almost the same content of lignin (13.1 and 12.1%, respectively) and both gave a higher yield than that of the CNF samples. This is due to the ability of the alkali treatment to effectively remove the non-cellulosic components, like lignin and hemicellulose [30]. However, as mentioned above, maleic acid could only partially hydrolyze hemicellulose due to its lower ionization degree than that of mineral acids [28,29]. Additionally, due to their lower content of α-cellulose, the yield of cellulosic solids prepared from parenchyma cells was lower than that from fiber cells. Furthermore, as determined by their different functions in bamboo, parenchyma cells contained more hemicellulose than fiber cells. Parenchyma is a vegetative tissue with a high content of organic substance and sugar, while the vascular bundle forms the internal skeleton of bamboo and provides adequate mechanical support.

### 3.2. Morphology Analysis

**Figure 3:**
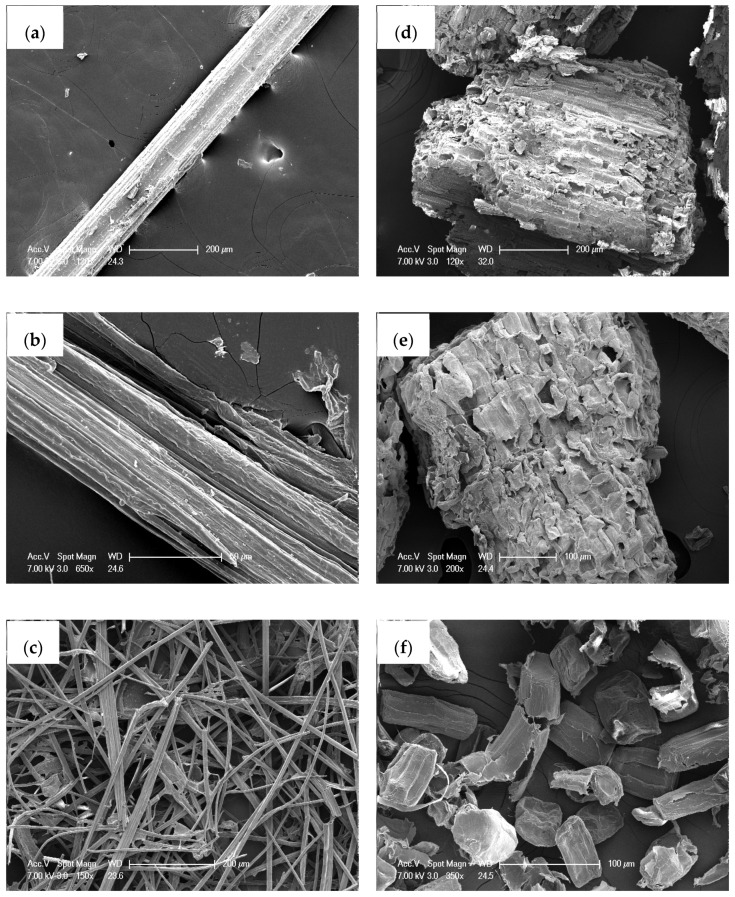
SEM images of fibers and parenchyma cells after chemical pretreatment. (**a**–**c**) F, F-LCSR and F-CSR, respectively; (**d**–**f**) P, P-LCSR, P-CSR, respectively.

The morphology of the fibers and parenchyma cells was compared between the LCSR and CSR samples (Figure 3). Fibers and parenchyma cells showed significant difference in cellular morphology, with the former being fibrous, and the latter being rectangular (Figure 3a,d). These two types of cellular morphology were probably formed as a result of the surface tension and pressure from surrounding cells. Compared with bamboo fiber cells, parenchyma cells have a much thinner cell wall and can be considered as feeble cells. After the maleic acid treatment, there were only some micro-cracks appearing on the surface of the LCSR samples (Figure 3b,e). In comparison, the alkali-treated CSR samples showed relatively significant alterations both in size and surface morphology. As shown in Figure 3c,f, due to lignin acting as an adhesive between cells [31], a number of single cells were obtained after the removal of lignin and hemicellulose. Moreover, the exposure of a large number of hydroxyl groups on the surface caused the contraction of microfilaments in the cell wall by hydrogen bonding [4], which flattened the CSR samples.

**Figure 4:**
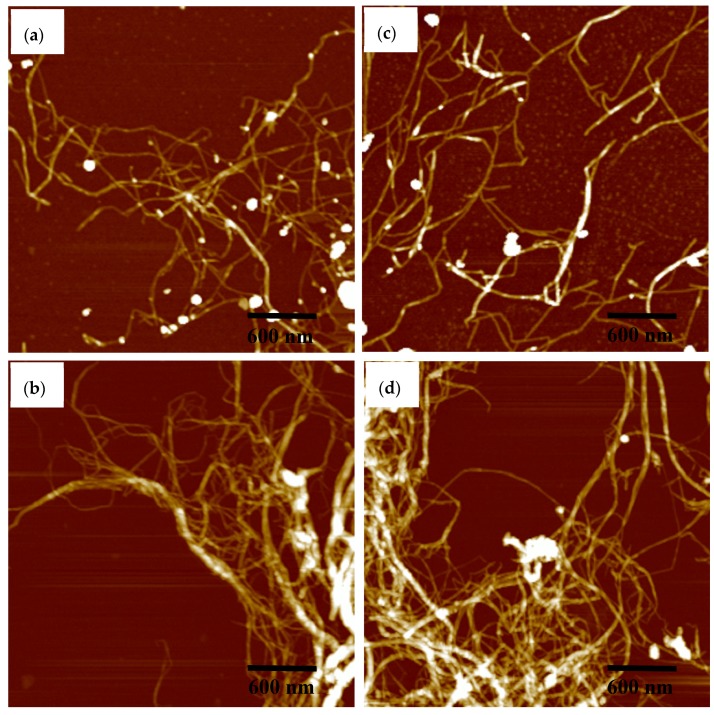
AFM height images of fibers and parenchyma cells after grinding. (**a**,**b**) F-LCNF and F-CNF, respectively; (**c**,**d**) P-LCNF, P-CNF, respectively.

The morphology of the LCNF and CNF samples, prepared by grinding three times, is shown in Figure 4. The images clearly show that the mechanical force, applied by grinding, destroyed the cell wall structures and effectively facilitated the separation of the fibril bundles in the samples into individual fibrils. Lignin particles were clearly visible in the LCNF samples (Figure 4a,c). Additionally, it is also notable that the diameter of LCNF is 20–40 nm, while that of CNF is only 10–20 nm, which is due to lignin acting to maintain the structural integrity of cellulose fibers by blocking access to them [32,33]. Despite their different shapes and structures, both the LCNF and CNF samples from fibers and parenchyma cells show a similar appearance of continuous nanofibrils aggregates.

### 3.3. X-ray Diffraction Analysis 

**Figure 5:**
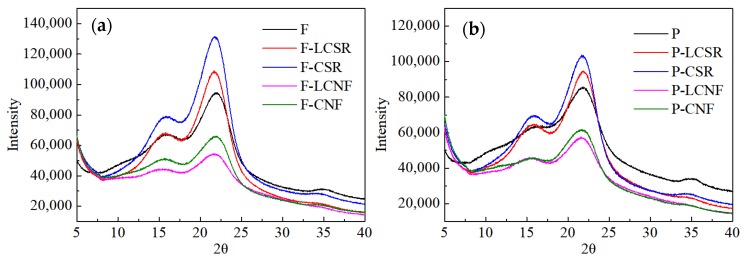
XRD patterns for the raw materials and their corresponding (L)CSR and (L)CNF samples from fibers (**a**) and parenchyma cells (**b**).

The CrI is an important factor that determines the elasticity, rigidity, thermal stability and absorption–desorption properties of the cellulose fibrils [34]. The XRD patterns for the original fibers and parenchyma cells followed by their corresponding (L)CSR and (L)CNF samples are displayed in Figure 5. All the samples showed sharp peaks at 2θ = 22.5° and 2θ = 16.5°, which corresponded to the (0 0 2) lattice plane of cellulose I [16,35]. This finding suggested that the methods, including the chemical pretreatment and grinding used in this work, did not destroy the crystal structure.

Although the crystal structure was not altered, the intensity of the diffraction peaks varied significantly among the samples. The CrI index values for various samples are listed in Table S1. As fibers require more α-cellulose to provide adequate mechanical strength as load-bearing units in bamboo, fibers have a much higher CrI compared with parenchyma cells. This result is consistent with the result of the analysis of the chemical composition. Compared with the three samples from different stages, the LCSR and CSR samples had the highest CrI. This is most likely due to the dissolution of a substantial amount of amorphous hemicellulose and lignin by the chemical pretreatment, which ultimately resulted in the overall increase in CrI of the (L)CSR. In addition, mechanical fibrillation by grinding seems to indiscriminately break up crystalline and amorphous regions of cellulose, resulting in a decrease in the CrI of the LCNFs and CNFs [36]. Such a breakage of the cellulose crystals is thought to contribute to the separation of its fiber bundles into nanofibrils [37]. Thus, the LCNF and CNF samples had the lowest CrI value among all the samples. Noteworthy, although the CrI values of the raw materials are quite different, the CrI values of the fibers and parenchyma cells subjected to the same chemical pretreatment and grinding process are similar.

### 3.4. Thermal Stability

**Figure 6:**
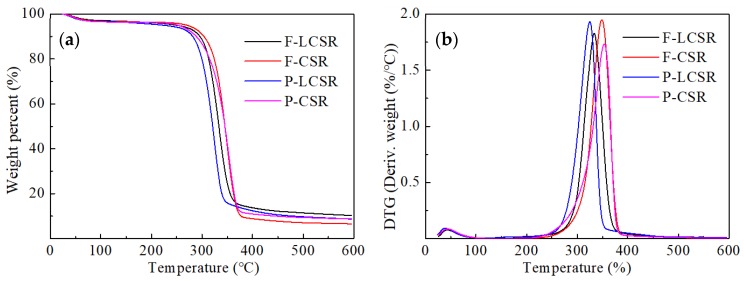
TGA (**a**) and DTG (**b**) for the samples from fibers and parenchyma cells.

The thermal stability of the LCSR samples was evaluated and compared with that of the CSR samples dried under the same conditions. The weight loss curve and derivative of the weight loss curve with temperature (dm/dT) were obtained (Figure 6). The onset degradation temperature, T_onset_, is defined as the temperature at which the sample weight loss becomes more apparent, and was obtained by the tangent method [10]. The maximal weight loss temperature, T_max_ is the maximum value of the derivative weight curve (dm/dT), and represents the temperature at which the decomposition is the fastest [31,38]. In the first stage, there was a very little weight loss from 25 to 100 °C due to the evaporation of water.

The T_onset_ and T_max_ of the samples at different stages are shown in Table S2. For the untreated materials, the thermal stability of fibers is much higher than that of parenchyma cells due to its lower hemicellulose content and higher CrI value, as mentioned above. The presence of amorphous hemicellulose, which is thermally less stable, can accelerate the degradation of fibers [10,39]. Another factor affecting thermal stability is the high cellulose crystallinity associated with larger crystallite size, which can act as barriers for the heat transfer and increase the fiber thermal stability [1]. On the basis of previous research, it was anticipated that residual lignin could improve the thermal stability of fibers [10,31]. However, in this study, the decomposition temperature of LCSR samples was lower than that of CSR samples. This result could be attributed to the higher retention of amorphous hemicellulose by the LCSR samples and their lower CrI than the CSR samples, which is consistent with the above analysis. In addition, there was no significant difference in the decomposition temperatures between the samples from fibers and parenchyma cells subjected to the same chemical pretreatment.

### 3.5. Water Contact Angle (WCA)

**Figure 7:**
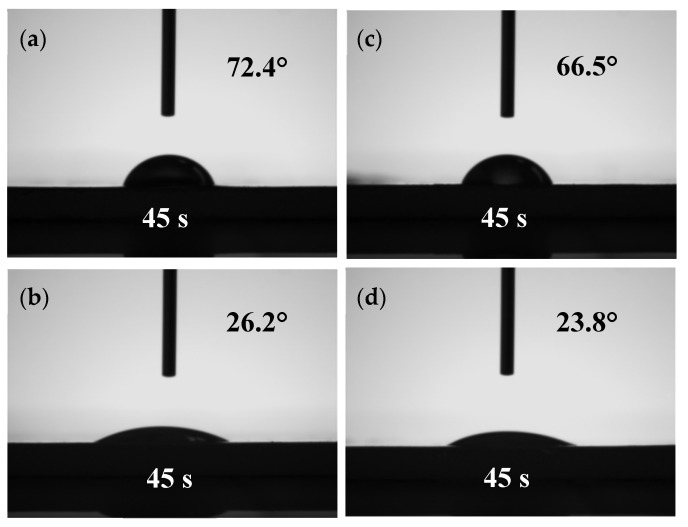
WCA (water contact angle) of LCNF and CNF films produced from fibers and parenchyma cells. (**a**,**b**) F-LCNF and F-CNF, respectively; (**c**,**d**) P-LCNF, P-CNF, respectively.

The WCA was measured to characterize the surface hydrophobicity of the films prepared from different samples as shown in Figure 7. For the films prepared from F-LCNFs and P-LCNFs, the WCA reached 72.4° and 66.5°, respectively; while for those prepared from F-CNFs and P-CNFs, the WCA were only 26.2° and 23.8°, respectively. These s results can be mainly explained by the difference in lignin content and the number of hydroxyl groups exposed. Lignin, in its native state, is more hydrophobic than the cellulose and hemicellulose, thus, it can act as a barrier for water penetration [13]. Additionally, the residual lignin may also be able to shield the accessible hydroxyl groups, thereby preventing water molecules from forming hydrogen bonds, which results in films with better hydrophobicity. Another influencing factor for the lower WCA of CNFs is the increase of porosity caused by the large-scale removal of lignin and hemicellulose.

### 3.6. Mechanical Testing

**Figure 8:**
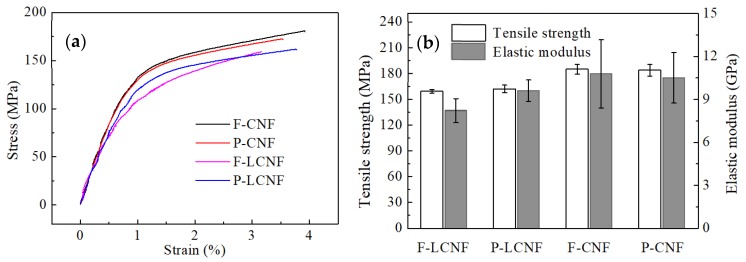
Typical tensile stress–strain curves (**a**) and the tensile strength and elastic modulus (**b**) of LCNF and CNF films produced from fibers and parenchyma cells.

Although it is quite difficult to perform a tensile test on individual nanofibrils, the tensile properties of the films prepared from (L)CNFs can be used to roughly evaluate the quality of nanofibrils from fibers and parenchyma cells [17]. We maintained the thickness of each film at a fixed value by using the same solid content. The results for representative curves of stress versus strain are presented in Figure 8a. Stress–strain curves of all the films displayed a typical bi-phase character, consisting of an initial linear segment followed by an obvious plastic one. The average ultimate tensile strength and Young’s modulus are shown in Figure 8b. Regarding LCNFs and CNFs, there were no significant differences in the mechanical properties of the films produced from fibers and parenchyma cells, especially in the Young’s modulus. Based on the combined results of the chemical composition and XRD analysis, it can be concluded that the nanofibrils isolated from bamboo fibers and parenchyma cells had broadly similar quality. The stress at break for films prepared from F-CNFs and P-CNFs was very similar at around 184 MPa, but this value decreased by about 13% for those prepared from F-LCNFs and P-LCNFs (160 MPa). According to related studies, mechanical properties, such as the modulus and strength of cellulose fibers, are directly proportional to the interfibril hydrogen bonds within the films [40]. However, the lignin hindered these hydrogen bonds and affected the mechanical properties of cellulose fibril-based films. Overall, the mechanical properties were not associated with the cell types but depended mostly on the chemical composition and CrI. 

## 4. Conclusions

In this study, a physical method based on density difference was used to separate fibers and parenchyma cells from mature Moso bamboo. We have successfully prepared the LCNFs and CNFs from each type of cell by a method involving chemical pretreatment combined with mechanical grinding. There were significant differences in morphology and structure between the untreated bamboo fibers and parenchyma cells, and the former showed lower content of hemicellulose and higher CrI than that of the latter. However, both the fibers and parenchyma cells can be successfully separated into nanofibrils which had similar chemical and physical properties. Additionally, the cellulose fibril-based films produced from fibers and parenchyma cells did not have significantly different mechanical properties. This finding suggests that although fibers and parenchyma cells play a different role in the growth of bamboo, they have less influence on the quality of LCNFs and CNFs. Moreover, LCNFs with a lignin content of 13% had better hydrophobicity than that of CNFs and still maintained significant tensile strength (160 MPa). Therefore, it can be predicted that the preparation of LCNFs is also an effective application of bamboo in nanocellulose. Accordingly, bamboo parenchyma cells can also be considered as a raw material for the production of nanocellulose (whether LCNF or CNF) like other plant fibers, which is beneficial to the full utilization of bamboo.

## Figures and Tables

**Table 1:** Abbreviations of the products obtained at each stage of sample preparation.

Samples	Treatment Conditions
F	Untreated fibers
F-LCSR	Fibers treated with maleic acid
F-LCNF	Fibers treated with maleic acid, grinding
F-CSR	Fibers treated with chlorite and KOH
F-CNF	Fibers treated with chlorite and KOH, grinding
P	Untreated parenchyma cells
P-LCSR	Parenchyma cells treated with maleic acid
P-LCNF	Parenchyma cells treated with maleic acid, grinding
P-CSR	Parenchyma cells treated with chlorite and KOH
P-CNF	Parenchyma cells treated with chlorite and KOH, grinding

**Table 2:** Comparison of the yield and chemical composition between fibers and parenchyma cells from bamboo.

Samples	α-Cellulose (%)	Hemicellulose (%)	Klason Lignin (%)	Total Yield (%)
F	44.6	29.4	23.6	100
F-LCSR	75.2	10.5	13.1	50.3
F-CSR	84.8	ND	ND	45.4
P	35.3	37.8	22.4	100
P-LCSR	72.7	13.0	12.1	42.8
P-CSR	84.5	ND	ND	36.3

ND: Not detected.

## Supplementary Materials

The following are available online at https://www.mdpi.com/1996-1944/13/1/237/s1, Table S1: CrI (%) of samples in different stage from fibers and parenchyma cells. Table S2: Tonset and Tmax values for samples from fibers and parenchyma cells.
