# Supplementary material for: A Comparison Study on the Characteristics of Nanofibrils Isolated from Fibers and Parenchyma Cells in Bamboo
Source: Materials (Basel). 2020 Jan 6;13(1):0. doi: 10.3390/ma13010237 (PMC6981400; doi:10.3390/ma13010237)
Supplement: Supplementary file 1 [file materials-13-00237-s001.pdf]

materials

MDPI

Article

A Comparison Study on the Characteristics of Nanofibrils Isolated from Fibers and Parenchyma Cells in Bamboo

Xiaofeng Zhang [1,2], Hanxiao Huang [1,2], Yan Qing [1], Hankun Wang [2,3,*] and Xingong Li[1,*]

1 College of Materials Science and Engineering, Central South University of Forestry and Technology, Changsha, 410004, China; zhangxf@icbr.ac.cn (X.Z.); huanghx@icbr.ac.cn (H.H.); qingyan0429@163.com (Y.Q.)

2 Institute of New Bamboo and Rattan Based Biomaterials, International Center for Bamboo and Rattan, Beijing, 100102, China

3 SFA and Beijing Co-built Key Lab for Bamboo and Rattan Science & Technology, Beijing, 100102, China

* Correspondence: wanghankun@icbr.ac.cn (H.W.); lxgwood@163.com (X.L.)

Table S1. CrI (%) of samples in different stage from fibers and parenchyma cells.

Samples	CrI (%)	Samples	CrI (%)
**F**	39.39	**P**	26.53
**F-LCSR**	42.55	**P-LCSR**	37.21
**F-CSR**	43.16	**P-CSR**	37.82
**F-LCNF**	23.78	**P-LCNF**	26.10
**F-CNF**	28.48	**P-CNF**	28.95

Table S2. T_{onset} and T_{max} values for samples from fibers and parenchyma cells.

Samples	T_{onset} (°C)	T_{max} (°C)
**F-LCSR**	313.44	332.82
**F-CSR**	327.43	349.07
**P-LCSR**	302.43	324.97
**P-CSR**	331.69	353.79
